# Understanding the Home Math Environment and Its Role in Predicting Parent Report of Children’s Math Skills

**DOI:** 10.1371/journal.pone.0168227

**Published:** 2016-12-22

**Authors:** Sara A. Hart, Colleen M. Ganley, David J. Purpura

**Affiliations:** 1 Department of Psychology, Florida State University, Tallahassee, Florida, United States of America; 2 Florida Center for Reading Research, Florida State University, Tallahassee, Florida, United States of America; 3 Florida Center for Research in Science, Technology, Engineering and Math, Learning Systems Institute, Florida State University, Tallahassee, Florida, United States of America; 4 Human Development and Family Studies, Purdue University, West Lafayette, Indiana, United States of America; Katholieke Universiteit Leuven, BELGIUM

## Abstract

There is a growing literature concerning the role of the home math environment in children’s math development. In this study, we examined the relation between these constructs by specifically addressing three goals. The first goal was to identify the measurement structure of the home math environment through a series of confirmatory factor analyses. The second goal was to examine the role of the home math environment in predicting parent report of children’s math skills. The third goal was to test a series of potential alternative explanations for the relation between the home math environment and parent report of children’s skills, specifically the direct and indirect role of household income, parent math anxiety, and parent math ability as measured by their approximate number system performance. A final sample of 339 parents of children aged 3 through 8 drawn from Mechanical Turk answered a questionnaire online. The best fitting model of the home math environment was a bifactor model with a general factor representing the general home math environment, and three specific factors representing the direct numeracy environment, the indirect numeracy environment, and the spatial environment. When examining the association of the home math environment factors to parent report of child skills, the general home math environment factor and the spatial environment were the only significant predictors. Parents who reported doing more general math activities in the home reported having children with higher math skills, whereas parents who reported doing more spatial activities reported having children with lower math skills.

## Introduction

The environment that parents provide at home can support children’s learning and development [1–3]. In particular, the home learning environment has been found to be a significant predictor of reading and math achievement [4–5]. A substantial body of research has examined the role of the “home literacy environment” (HLE), a term used to describe literacy activities children engage in with their parents [6–10]. Despite ample evidence supporting the importance of the HLE, far less research has examined the relation of the “home math environment” (HME; note, this is often called the more narrow term “home numeracy environment” in the literature, but HME is used in this study as this construct is approached from a broader perspective). Drawing parallels to the HLE, the HME consists of activities which parents and their children engage in which are intended to support mathematical development either through direct (e.g., counting, number naming) or indirect (e.g., cooking, playing store) activities [11]. Critically, parents have reported enjoying literacy activities more than math activities [12], suggesting that the home math environment might be a place in need of more attention.

The research in this area has indicated that what parents do with their children mathematically, and how often they do it, is predictive of their child’s mathematical ability [11, 13–15] L. In the present study we add to this body of literature by examining the measurement structure of the home math environment, as well as its relation to parents’ reports of children’s math skills. Importantly, we consider multiple potential alternative explanations for the relation between the HME and children’s skills in our analyses as well (socioeconomic status, parent math anxiety, parent math skills).

### Measurement Structure of the Home Math Environment

The HME has been measured in a variety of ways. For example, many have simply measured all aspects of the home environment thought to be related to math development, thereby considering the HME as a unidimensional construct [13, 16]. In contrast, LeFevre and her colleagues have advanced a model, where the home math environment is made up of two aspects, direct and indirect numeracy activities [6, 17]. “Direct numeracy activities” include specific number tasks that parents engage in with their children for the purpose of explicitly teaching quantitative skills, such as teaching number names or counting. “Indirect numeracy activities” include a wider range of real-world tasks that parents engage in with their children that likely have an incidental relation to quantitative skill development, such as cooking or playing cards [11].

Another multidimensional model has been proposed with two separable aspects of the home environment related to math development: math activities and spatial activities [18]. “Math activities” were those related to numerical activities that would likely develop arithmetic skills, such as playing card games that use numbers or counting. “Spatial activities” were those that were related to activities that were likely to develop spatial skills, considered a precursor to geometry and measurement skills, such as playing with puzzles, measuring, and building [14, 18]. To the best of our knowledge, no one has formally tested the full range of possible models of the HME to gain a better understanding of the measurement structure of the construct (see [17], for principal component analyses testing the separation of advanced formal numeracy and basic formal numeracy home practices). We test different ways of conceptualizing the HME found in the literature, and fit a series of confirmatory models to determine the best fitting model of the home math environment.

### Relation between the Home Math Environment and Math Skills

Regardless of how it is defined, there have been inconsistent findings thus far concerning the role of the home math environment and children’s math performance. In some past work, exposure to numbers in daily family life was related to the development of mathematical abilities [19], and engaging with number-related activities was related to performance on a standardized measure of mathematics ability [13]. Cross-cultural work in European American, Chinese American, and Taiwan Chinese families has found a positive relation between numeracy activities and formal math skills [20–22] (. A recent study found that home activities that encouraged numerical performance predicted performance on tasks measuring the Exact Number System, but not the Approximate Number System [23].

Work that separately examines distinct aspects of the home math environment has found mixed results in regard to the importance of each type of numeracy activities for children’s math skills. In work that examines direct and indirect numeracy activities, direct numeracy activities have been shown to be associated with numeracy skills [16, 17, 24–26]; other work showed no relation between the direct numeracy activities and math performance [11, 27, 28]. For indirect numeracy activities, there is some support for the association between these activities and math performance [11, 17], but this association has been examined less than the association between direct numeracy activities and math performance. Turning to the work looking at math activities versus spatial activities, Dearing and colleagues[18], in a sample of all girls, found that the home math environment, but not the home spatial environment, predicted children’s arithmetic performance, and neither predicted children’s spatial performance.

### Relation of Home Math Environment to Children’s Math Skills, Accounting for Potential Alternative Explanations

As with all areas of child development, the development of math skills is complex and therefore it is critical that the relation between the home math environment and math skills be considered within the context of other important factors, including socioeconomic factors and internal parent characteristics. Socioeconomic factors have been highlighted when considering the role of the home math environment in child math performance. Although often included as a control variable only [e.g., 17], research does show that homes with fewer socioeconomic resources tend to have less stimulating home environments [29–30]. Further, Dearing and colleagues [18] found that children with less family economic strain and mothers with more education had richer general home learning environments, which included participating in more numerosity and spatial activities. Other research also finds that socioeconomic factors are directly predictive of children’s cognitive development (i.e. not solely mediated through the home learning environment). A meta-analysis indicated the average effect size (*r*) of the association between socioeconomic status (SES) and math achievement was .35 [31].

Internal parent factors have also been associated with the home math environment. For example, higher parent academic expectations have been linked to more frequent numeracy activities in the home and math performance [24–25]. A parent characteristic that has received considerable recent attention in the broader literature, but not in studies considering the home math environment, is parent math anxiety [32–35]. Parents’ own anxiety about doing math has been linked with children’s math performance, although the relation is not always direct. For example, high parent math anxiety was negatively associated with children’s math performance when children also had higher math anxiety [33]. Specifically, when both fathers and sons have high math anxiety, the sons’ GPA is lower than if either the son or father had low math anxiety [33]. Another recent study found that parents with high math anxiety who frequently help their children with their homework had children with lower math performance, indicating a possible link between parent math anxiety, the home math environment, and math performance [34]. Experimental work showed that when children who had parents with high math anxiety interacted with a math app, the children had higher math performance than similar children who did not interact with the math app, and this effect was non-significant for parents with lower levels of math anxiety [32]. Thus, similar to the idea that parents who struggle with reading are likely to not have many books in the home or read at home themselves [36], it is likely that parents with high math anxiety have homes where they engage in fewer activities related to math, and that perhaps a math app was effective with these parents because it worked as a springboard for other positive conversations and activities about mathematics in the home. Overall, if parents are anxious about math, it is possible that the interactions they have at home that involve math are not positive experiences for either the parent or the child. One possible mechanism for this relation is parent talk about number and spatial features. Previous work has found that during normal home activities, parent talk about number and spatial features was related to child numerosity and spatial skills [37–39].

Another key reason why internal parent factors are important to consider is because parents pass on genes for math skills to their children as well as provide the home math environment for their children. Therefore, it is likely that children’s genes for math skills are correlated with their home environment, a gene-environment correlation [40–41]. Evidence from twin studies has shown that there is a moderate genetic influence on different aspects of math performance [42]. Genetically-sensitive designs are the only way to fully disentangle these gene-environment correlations from the direct effect of the home math environment on math skill development. However, when these data are not available, accounting for parent math skills when looking at the relation between the home math environment and children’s math skills allows for a proxy control of gene-environment correlations. Although occasionally parent skill is included in analyses when examining the association of the home math environment on children’s math performance [18], it is not common. When including maternal spatial skills as a predictor of the general home learning environment, as well as children’s numerosity and spatial performance, Dearing et al. [18] found that maternal spatial skills directly predicted children’s numerosity and spatial performance, but not indirectly through the home environment. This highlights the importance of including parent’s own math skills, as this finding points to the direct relation of parent’s skills to their children’s skills, partially due to shared genetics. It could also be the case that parent’s skills are indirectly related to children’s skills through the home environment such that parent skills are associated with the home environment they set up for their children, and then this environment predicts child skills. Follow up work of the Dearing et al. [18] study suggested that the link between maternal spatial skills and children’s numerosity and spatial performance was mediated by maternal supportiveness, indicating a potential environmental explanation [43].

### Current Study

Given the growing literature on the home math environment, there were three goals of the current study, which included a large online sample representing a diverse sample of parenting styles in US homes. The first goal was to identify the best factor structure of the home math environment. It has been suggested that the reason the literature is mixed on the association of HME with math skills is because significant effects are indicated only when the HME is measured using a refined instrument based on an explicit theoretical model [17]. Given the lack of agreement in the literature, we asked parents about activities that could be considered direct and indirect numeracy environment activities, as well as number and spatial activities. We then sought to explicitly test the different ways to conceptualize the home math environment, either as a singular general factor, or following LeFevre and colleagues with direct and indirect (both of which include both number and spatial activities), or following the conceptualization of number and spatial home environments (both of which could be direct and indirect activities; i.e. [18]). Different factor models were tested to determine the best fitting model for these data.

After determining the best fitting home math environment structure, our second goal was to examine the role of the home math environment in predicting children’s math skills, as reported by their parents, while controlling for child age and gender. Math skills included both numeracy and spatial skills as both have been linked to school-based math performance (e.g., [44]). With this research question, we contributed to the growing literature exploring the role of the HME in predicting children’s math skills (although here measured as parent report of children’s math skills).

We then build off the second goal to address our third goal, which was to examine the role of HME in predicting parent report of children’s math skills after including other factors which could be important in understanding any possible association between the HME and parent report of children’s math skills. In other words, we explore whether any relation between the home math environment and parent report of child’s skills remains after accounting for possible alternative explanations. First, we considered the role of household income as both a direct predictor of parent report of children’s math skills, and an indirect predictor of parent report of children’s math skills through the HME. Household income, a proxy for socioeconomic status, has reliably been linked with children’s math skills and the home learning environment. It could be serving as a “missing variable” that, when excluded, makes it appear that there is a relation between HME and child math skills, but once this variable is included any relation might be attenuated if it is truly SES that is the critical variable (and SES is correlated with HME). Second, in line with the methods we used for including SES in the models, we included parent math anxiety, adding it as a direct predictor of parent report of children’s math skills, and as an indirect predictor of parent report of children’s math skills through the HME. Parent’s math anxiety has only recently been examined in the literature but there is potential for an association with children’s math skills and with the HME. In a third model, we included parent’s own math skills as a direct and indirect predictor. Importantly, parents not only provide the home math environment for their children, but also genes related to math performance. Therefore, any correlation found between the HME and children’s math skills, without controlling for known genetic influences on math skills, may be confounded by gene-environment processes. By including parent’s own math skills within the broader model (as a proxy control for gene-environment processes), we will explicitly test the role of HME in predicting parent report of children’s math skills, outside of any genetic confounds. Finally, in a fourth model, we tested all variables, and their direct and indirect relations, in one model, to examine the relation between the home math environment and parent report of child math skills, after all variables are accounted for.

## Materials and Methods

### Participants

Participants in the study were parents of children ages 3 to 8. The initial sample included 355 participants from Mechanical Turk (www.mturk.com). Participants were required to have at least one child who was between the ages of 3 through 8, be at least 18 years old, English-speaking United States residents, have completed at least 1000 HITs, and have a HIT approval rate of equal to or greater than 98%. After surveying the data, 16 participants were dropped, one because it was clear he or she simply selected the lowest value for all variables and 15 because their reported child age was outside of the age required to participate. Therefore, the final sample size for analysis was *n* = 339. The sample included 66.34% female (*n* = 235) participants with a mean age of 34.45yrs (*SD* = 6.86yrs). When reporting ethnicity, 6.30% (*n* = 22) identified as being Hispanic/Latino. When separately reporting race, 80.83% (*n* = 274) identified their race as White, 8.26% (*n* = 28) as Black or African American, 7.37% (*n* = 25) as Asian, and the remainder as Mixed race or other. Most of the participants described the area they lived as “suburban or small city” (58.41%, *n* = 198), with 25.96% “urban or large city” (*n* = 88), and the remaining in a “rural” location. The current household income mode was $30,000-$49,000 and median was $50,000 –$69,000. Most participants had either completed some college (24.19%, *n* = 82) or graduated from a 4-year college (35.10%, *n* = 119), with a further 11% having graduated high school or equivalent (*n* = 38) and 12% having completed graduate or professional school (*n* = 41). Fifty-five percent of the participants reported that the child they were reporting on was female (*n* = 187), and the mean age of the children the participants were reporting on was almost six years old (*M* = 5.96, *SD* = 1.63). Compared to the 2015 Census of married, cohabiting or single parent households with a child under 18yrs, the current sample was roughly equivalent for race (76% White, 15% Black, 5% Asian), slightly underrepresented for Hispanic/Latino (19% Hispanic/Latino), and slightly better educated (21% high school graduate, 29% some college or AA degree, 22% Bachelor’s degree, 18% professional or graduate degree; (http://www.census.gov/hhes/families/data/cps2015H.html).

### Procedures and Measures

All aspects of this study were approved by the Florida State University Institute Review Board in accordance with the Declaration of Helsinki (HSC No. 2015.14562). Prior to completing the questionnaire, participants were asked to read an Informed Consent Form and give consent by clicking “Agree”. Participants completed the measures in the following order: questions about their child in the specified age range (they were instructed to select one within the range if they had multiple children of the appropriate age), questions about their own ability, the math survey, the home environment survey, approximate number system (participants navigated to panamath.com and complete the task), and demographic questions. The questionnaire took approximately 30 minutes to complete. Participants were paid $.50 for their time. All data from the full data collection (i.e. including the measures not used in the present analyses), including a copy of the survey administered, are available publically in the Supporting Information file, S1 Data, S1 File.

### Measures

#### Home Environment Survey

The Home Environment Survey was an investigator created 52-item survey concerning frequency of math, spatial, and reading activities (many items came from [11]and [18]). The participant was instructed “ON AVERAGE, how often do you do each of the following with your child outside of school? We are trying to determine what children and their parents do inside the home that might be related to children’s math achievement in school. For each activity below, please select the option that indicates how often you did each.” The items followed, with a Likert-type scale choice of 1 to 6 (“Never”, “Monthly or less”, “Less than once a week, but a few times a month (1–3 times)”, “About once a week”, “A few times a week (2–4 times)”, or “Almost daily”). There were 48 items reporting activities that might be related to the home math environment (see Table 1 for the items). The remaining four items were related to the home literacy environment, and were not included in these analyses.

**Table 1:** Response rates, factor membership, factor loadings, and descriptive statistics for all home math environment items.

#	Item	% responding “Never”	Direct or Indirect^a^	HNE Or HSE^a^	Direct, Indirect or Spatial^a^	General Home Math Environment Factor loadings^b^	Direct Numeracy Environment Specific Factor loadings^b^	Indirect Numeracy Environment Specific Factor loadings^b^	Spatial Environment Specific Factor loadings^b^	Mean	*SD*	Skew
**Items included in the final model**												
**Direct Numeracy Environment Specific Factor**												
2	Identify names of written numbers	6.20	Direct	HNE	Direct	0.41	0.64	—	—	4.16	1.50	-0.54
3	Play with numerical magnets	24.78	Direct	HNE	Direct	0.50	0.29	—	—	3.09	1.68	0.19
4	Counting objects	3.83	Direct	HNE	Direct	0.33	0.80	—	—	4.74	1.42	-1.01
5	Sort things by color, shape, or size	4.72	Direct	HNE	Direct	0.51	0.58	—	—	4.27	1.40	-0.61
6	Count down (10, 9, 8, 7…)	5.31	Direct	HNE	Direct	0.51	0.58	—	—	4.27	1.45	-0.58
8	Printing numbers	5.01	Direct	HNE	Direct	0.49	0.44	—	—	4.25	1.42	-0.62
17	Use number activity books	6.19	Direct	HNE	Direct	0.69	0.26	—	—	3.86	1.42	-0.32
18	Read number storybooks	7.67	Direct	HNE	Direct	0.62	0.35	—	—	3.76	1.45	-0.25
41	Note numbers on signs when driving or walking with children	11.50	Direct	HNE	Direct	0.55	0.34	—	—	3.94	1.65	-0.40
48	Recite numbers in order	7.12	Direct	HNE	Direct	0.31	0.60	—	—	4.53	1.52	-0.99
**Indirect Numeracy Environment Specific Factor**												
9	Talk about money when shopping (e.g., “which costs more?”)	7.96	Indirect	HNE	Indirect	0.51	—	0.60	—	3.97	1.43	-0.59
10	Measure ingredients when cooking	7.67	Indirect	HNE	Indirect	0.47	—	0.64	—	4.04	1.44	-0.55
11	Being timed	13.86	Indirect	HNE	Indirect	0.45	—	0.45	—	3.57	1.56	-0.19
13	Making collections	14.16	Indirect	HNE	Indirect	0.61	—	0.17	—	3.40	1.50	-0.09
15	Using calendars and dates	6.19	Indirect	HNE	Indirect	0.53	—	0.35	—	4.13	1.49	-0.44
20	Play card games	8.26	Indirect	HNE	Indirect	0.55	—	0.18	—	3.65	1.36	-0.42
24	Interact with clocks (such as pointing out to your child where the big hand and the little hand on the clock are and discussing what time it must be)	7.96	Indirect	HNE	Indirect	0.56	—	0.23	—	4.09	1.53	-0.54
42	Use numbers when referring to temperatures, time, and dates	5.01	Indirect	HNE	Indirect	0.42	—	0.23	—	4.61	1.45	-0.98
**Spatial Environment Specific Factor**												
27	Draw maps (such as treasure hunt maps)	22.42	Indirect	HSE	Spatial	0.54	—	—	0.61	2.85	1.47	0.36
28	Draw plans for houses, forts, castles, or other buildings or layouts	28.94	Indirect	HSE	Spatial	0.50	—	—	0.81	2.65	1.48	0.52
29	Measure the length and width of things	20.94	Indirect	HSE	Spatial	0.64	—	—	0.37	2.91	1.42	0.22
30	Use kits to build models (such as airplanes, animals, dinosaurs, doll houses)	17.40	Indirect	HSE	Spatial	0.54	—	—	0.40	2.79	1.37	0.49
39	Fold or cut paper to make 3D objects (such as origami, paper planes)	25.96	Indirect	HSE	Spatial	0.63	—	—	0.14	2.74	1.50	0.49
**Items not included in the final model**												
1	Use number or arithmetic flashcards	17.11	Direct	HNE	Direct	—	—	—	—	3.14	1.53	0.16
7	Learning simple sums (i.e. 2+2 = 4)	4.42	Direct	HNE	Direct	—	—	—	—	4.22	1.38	-0.58
12	Playing with calculators	19.17	Indirect	HNE	Indirect	—	—	—	—	3.21	1.58	0.08
14	“Connect-the-dots” activities	9.73	Indirect	HNE	Indirect	—	—	—	—	3.52	1.36	-0.20
19	Play board games with a die or spinner	7.37	Indirect	HNE	Indirect	—	—	—	—	3.65	1.33	-0.48
21	Use computer or video games to do drawing or painting or matching and playing with shapes	5.01	Direct	HSE	Spatial	—	—	—	—	4.24	1.44	-0.61
22	Uses a computer or video games to do addition, subtraction, or other math activities	10.62	Direct	HNE	Direct	—	—	—	—	3.82	1.58	-0.38
23	Uses a computer or video games to do spatial tasks (such as the game Tetris)	12.39	Direct	HSE	Spatial	—	—	—	—	3.65	1.58	-0.21
25	Count out money	7.67	Direct	HNE	Direct	—	—	—	—	3.96	1.43	-0.49
26	Play with puzzles (such as picture puzzles, tangrams, slide puzzles, 3D puzzles)	3.24	Indirect	HSE	Spatial	—	—	—	—	4.19	1.25	-0.59
31	Guess the number of things (such as candies in a jar)	22.71	Indirect	HNE	Indirect	—	—	—	—	2.83	1.45	0.37
32	Add or subtract numbers in your head with your child	15.34	Direct	HNE	Direct	—	—	—	—	3.72	1.63	-0.34
33	Compare the sizes of numbers (such as 5 is more than 4)	6.78	Direct	HNE	Direct	—	—	—	—	4.09	1.47	-0.48
34	Play with Legos or other building blocks	1.77	Indirect	HSE	Spatial	—	—	—	—	4.78	1.23	-1.05
36	Keeping track of money with a Piggy Bank	26.84	Indirect	HNE	Indirect	—	—	—	—	3.14	1.73	0.13
43	Learn and sing math songs (such as Schoolhouse Rock)	22.71	Direct	HNE	Direct	—	—	—	—	3.18	1.68	0.17
44	Do math word problems	15.98	Direct	HNE	Direct	—	—	—	—	3.52	1.62	-0.17
45	Games in the car that involve counting and/or math	14.45	Direct	HNE	Direct	—	—	—	—	3.62	1.59	-0.25
46	Helping with math homework	13.06	Direct	HNE	Direct	—	—	—	—	4.46	1.74	-0.96
	**Items not included because of lack of response**											
16	Have your child wear a watch	46.90	—	—	—	—	—	—	—	2.55	1.82	0.76
35	Play with an abacus	60.47	—	—	—	—	—	—	—	2.01	1.49	1.25
37	Play with dominos	36.58	—	—	—	—	—	—	—	2.49	1.49	0.63
38	Use scales	40.12	—	—	—	—	—	—	—	2.36	1.45	0.73
40	Play with a math mat	53.39	—	—	—	—	—	—	—	2.22	1.60	0.97
47	Doing math in reference to sports (calculating batting averages, etc.)	42.14	—	—	—	—	—	—	—	2.65	1.76	0.59

*Note*. All factor loadings significant at *p* < .05. HNE = home numeracy environment; HSE = home spatial environment; Direct = direct numeracy environment; Indirect = indirect numeracy environment; Spatial = spatial environment. *N* = 339. Range of responses = 1–6.

^a^These columns represent the factor which each item was loaded onto while testing the different home math environment models

^b^These columns represent the factor loadings from the final home math environment model

#### Household income

Participants were asked to select “current household income”, based on an investigator created scale. There were 12 total choices, “less than $10,000”, “$10,000–29,000”, “$30,000–49,000” and so forth to “210,000 or more”. Parents could also report “Don’t know” or “Don’t want to say”, which were subsequently coded as missing.

#### Parent math anxiety

Parent math anxiety was measured using six-items from the Gulick Math Anxiety Scale [45]. The original scale included eight items, but the two items that involved situations specific to being a student were not used. Participants were told “Some individuals feel anxiety when in certain situations involving mathematics. Please rate your level of anxiety when considering the following situations:”, and then asked to rate their anxiety for each item on a 5-point Likert scale (from 1 to 5, “low anxiety” to “high anxiety”). Sample reliability was high, Cronbach’s alpha = .89.

#### Parent Approximate Number System (ANS)

For parent numerosity, participants were instructed to navigate to http://panamath.org/expt5_fsu/, which is a link specifically set up for the present investigator team to use by the Panamath.org team. Panamath is an online test measuring the ability of an individual to nonverbally represent number, or understand and manipulate numerical quantities non-symbolically [46–48]. The process underlying this ability of non-symbolic number sense is called the Approximate Number System (ANS), an intuitive sense of number. After entering their age and practicing, participants were shown brief displays (600ms) of intermixed blue and yellow dots with five to 20 dots per color, and asked to determine if there were more blue (by pressing the “b” key”) or yellow dots (by pressing the “y” key). In total, the participants were given 120 trials of various ratios of dot quantities (~5-7min of testing time), and accuracy and response time for each trial was recorded. Panamath then calculates the participant Weber fraction (*w*-score), which represents the smallest ratio that can accurately be discriminated by a given individual, and reports it in a hyperlinked pdf file. The participant was asked to provide the hyperlink in the survey, from which the *w*-score was obtained. Due to the additional step of having to navigate to a different website and then copy the hyperlink in Qualtrics, 19 participants did not report *w*-score data (*n* = 330 participants with ANS data). Mirroring Halberda et al.[49], an additional six participants had their *w*-score data set to be missing for having *w*-scores greater than four standard deviations from the sample mean.

#### Covariates

Parent-reported child gender and child age, and parent gender, were included as covariates. Gender was recorded as “boy” (coded as “1”) or “girl” (coded as “2”). Age was recorded by parent-reported date of birth, and age was calculated as years to September 1, 2015 (i.e. a date approximately in the middle of data collection and that corresponds to a common cutoff date for school entry in the US). Parent gender was recorded as “male” (coded as “1”) and “female” (coded as “2”).

#### Parent report of child skills

Parent report of child math skills were captured through two methods and five measures. For the first method the participant was asked to rate his or her child’s skills, “Compared to 100 people this child’s age, he/she would be better than ________ of them with math”, “Compared to 100 people this child’s age, he/she would be better than ________ of them with numbers, such as counting or knowing number names” and “Compared to 100 people this child’s age, he/she would be better than ________ of them with spatial skills, such as doing a puzzle”. The participant was then asked to move a slider to select a response between 0 and 100. The number selected between 0 and 100 was used as the score for “child math skill”, “child number skill”, and “child spatial skill” respectively.

As a second parent report of children’s math skills, parents filled out the Colorado Learning Disabilities Questionnaire (CLDQ;[50]), which is a 20-item parent report rating scale developed to be a brief screening measure for learning difficulties. Three items from the questionnaire relate to math difficulties, and four items related to spatial difficulties which parents rate on a 5-point Likert scale (“never/not at all” to “always/a great deal”). Example items from the math difficulties scale include “Was/is your child worse at math than at reading and spelling?” and “Does/did your child have trouble learning new math concepts such as carrying or borrowing?” The three items were combined into a sum score representing “child CLDQ math”. Reliability for this scale in this sample is adequate, Cronbach’s alpha = .85. Example items from the spatial difficulties scale include “Is/was your child’s handwriting spatially disorganized?” and “On arithmetic problems, does/did your child have difficulty keeping the numbers lined up in columns?” The four items were combined into a sum score representing “child CLDQ spatial” and used for analyses. Reliability for this scale in this sample is adequate, Cronbach’s alpha = .81.

### Analyses

After descriptive statistics were calculated, it was determined that the *w*-score was heavily skewed, so it was log-transformed. Subsequently, the CLDQ and *w*-score data were reverse scored (by multiplying by -1), so that higher scores represented “better” performance. Afterwards, all raw data, other than parent and child gender, were standardized to a *z*-score distribution to assist with model fitting. Data management, descriptive statistics, and data transformations were done in SAS 9.4 (code is available publically in the Supporting Information file, S1 Code).

#### Factor structure of the HME

To address the first research question, a series of eight confirmatory factor models were run to determine the best fitting model of the home math environment. A singular “home math environment” factor model was fitted, representing all possible activities in the home. Subsequently, two two-factor models were run. The first two-factor model represented the “direct numeracy environment”, with all items related to activities meant to explicitly teach children quantitative skills, and the “indirect numeracy environment”, with all items associated with activities where the potential for mathematical engagement was present, but not necessarily the central focus. The second two-factor model represented the “home numeracy environment” (HNE), with all items relating to numeracy activities, and the “home spatial environment” (HSE), with all items relating to spatial activities. Finally, after observing the very high correlation between the factors in the two two-factor models (*r* > .80), two bifactor models were considered. A bifactor model allows for data to be represented with both unidimensional (e.g., a single common home math environment factor) and multidimensional latent structures (e.g., subsets of items that tap similar content domains, like indirect numeracy environment activities). In these models, all items were loaded onto a general “home math environment” factor, and then for the first bifactor model, the residual variance was modeled into specific factors representing “direct numeracy environment” and “indirect numeracy environment”, and for the second bifactor model, specific factors representing the “home numeracy environment” and “home spatial environment”.

After considering these first five models, which were based directly on the previous literature, it was determined that there were other alternative models that we should consider, specifically a three-factor model, a three-factor bifactor model, and a four-factor model. The three-factor model represented the “direct numeracy environment”, “indirect numeracy environment” and the “spatial environment”. The three-factor bifactor model was modeled as a general “home math environment”, and then three specific factors representing the same three factors as listed in the three-factor model. The four-factor model represented “direct numeracy environment”, “indirect numeracy environment”, “direct spatial environment” and “indirect spatial environment”. As a note, it was not possible to test a bifactor version of the four factor model, as the “direct spatial environment” had only 2 items, and with the uncorrelated factors in the bifactor model this was not identified. The selection of which item would be loaded onto which factor was done based on the past literature, as well as by agreement across the investigators (see Table 1 for this information about each item).

#### Relation of HME to parent report of child skills

After the best fitting factor model for the home math environment was determined, it was used within a broader structural equation model to determine the role of the home math environment in predicting children’s math skills. Child and parent gender and child age were included as covariates.

#### Relation of HME to parent report of child skills, accounting for potential alternative explanations

After the structural equation model representing the prediction of HME to parent report of child skills was finalized, four models were run to test potential alternative explanations of the relation between the HME and parent report of child skills. The first included household income as a direct and indirect (via the HME) predictor of parent report of child skills. The second included parent math anxiety as a direct and indirect predictor of parent report of child skills. The third model included parent math skills as a direct and indirect predictor of parent report of child skills. The fourth model included all possible potential alternative explanatory variables in the model as direct and indirect predictors of parent report of child skills.

For all models, model fit was evaluated using multiple indices, including chi-square (*χ^2^*), comparative fit index (CFI), Tucker-Lewis index (TLI), root mean square error of approximation (RMSEA), and standardized root-mean-square residual (SRMR). Traditionally, CFI and TLI values equal or greater than .95, RMSEA values below .08, and SRMR values equal or less than .05 are preferred for an excellent model fit [51]. The *χ^2^* difference test, Akaike information criterion (AIC) and sample size-adjusted Bayesian information criterion (BIC) were used to evaluated relative model fit. A significant *χ^2^* difference test suggests that the model with more degrees of freedom (i.e. more constrained) provides a worse fit to the data than the model with fewer degrees of freedom (i.e. less constrained). Additionally, lower AIC and BIC values indicated a better fitting model. The measurement modeling and subsequent structural modeling were conducted in Mplus 7.31 using a maximum likelihood estimator [52]. Mplus code is available publically in the Supporting Information file (see S2–S18 Codes, S2 and S3 Data).

## Results

### Factor Structure of the Home Math Environment

All descriptive statistics are displayed in Table 1. The first goal of this work was to examine the structure of the home math environment. Before beginning to fit the different models, items which had greater than one-third of the sample responding “never” were dropped from all modeling. This resulted in six items (16, 35, 37, 38, 40 and 47) being dropped (see Table 1). The fit statistics of the eight different models are displayed in Table 2. In general, the criterion-based fit indices (i.e. CFI, TLI, RMSEA, and SRMR) suggested that none of the models provided an excellent fit to the data (i.e. based on[51]). As the factors were chosen based on a review of the literature, the relatively best fitting model was selected based on the relative fit indices (χ^2^, AIC and BIC). Based on these indices, the bifactor model with direct numeracy environment, indirect numeracy environment, and spatial environment specific factors was favored, as it had the lowest AIC and BIC values.

**Table 2:** Model fit indices from testing the various factor models representing the home math environment.

Model	*χ^2^*	*df*	*P*	AIC	Adj BIC	RMSEA	RMSEA lower bound	RMSEA upper bound	CFI	TLI	SRMR
1 Factor: Home Environment	3903.53	819	.00	35980.40	36062.78	.105	.102	.109	.60	.58	.09
2 Factor: Direct and Indirect	3667.04	818	.00	35745.91	35828.95	.101	.098	.105	.63	.61	.09
Bifactor: Direct and Indirect	2668.54	777	.00	34829.40	34939.24	.085	.081	.088	.75	.73	.07
2 Factor: HNE and HSE	3734.97	818	.00	35813.84	35896.88	.103	.099	.106	.62	.60	.10
Bifactor: HNE and HSE	2653.58	777	.00	34861.19	34971.03	.084	.081	.088	.75	.73	.07
3 Factor: Direct, Indirect & Spatial	3570.61	816	.00	35653.47	35737.82	.100	.096	.103	.64	.62	.10
Bifactor: Direct, Indirect & Spatial	2542.20	777	.00	34703.07	34812.91	.082	.078	.085	.77	.75	.07
4 Factor: Direct numeracy, Indirect numeracy, Direct spatial, Indirect spatial	3416.58	813	.00	35505.44	35591.75	.097	.094	.101	.66	.64	.09

*Note*. Direct = direct numeracy environment; Indirect = indirect numeracy environment; Spatial = spatial environment; HNE = home numeracy environment; HSE = home spatial environment.

After the bifactor model with a general home math environment factor and direct numeracy environment, indirect numeracy environment, and spatial environment specific factors was selected as the best fitting model, we conducted a series of exploratory model fitting steps to improve overall model fit. This was done because we intended to use this model in further structural equation modeling in the second and third research goals, we wanted a better fitting model. Modification indices suggested many correlated residuals should be included for better fit, and this, along with the very high alpha (alpha for full HME questionnaire was .95) and high item-total correlations for the full scale, suggested the poor fit may be due to having too many highly related items. Therefore, we decided to entirely drop items from the model that were either not significantly loading onto one of the factors in the bifactor model, or that were negatively loading onto a factor. This decision was made in keeping with psychometric theory (e.g., negatively loaded items suggest misinformation on the construct, in that responding doing the activity more was associated with HNE scores).

These initial step of item dropping resulted in 17 items being dropped, four for nonsignificant loadings onto the direct numeracy environment specific factor (items 1, 7, 33, and 45), five for significant but negative loadings onto the direct numeracy environment specific factor (items 22, 25, 32, 44, and 46), four for nonsignificant loadings onto the indirect numeracy environment specific factor (items 12, 14, 31, and 36), three for nonsignificant loadings onto the spatial environment specific factor (items 23, 26 and 34), and one for a significant but negative loading onto the spatial environment specific factor (item 21). After running the bifactor model with these items dropped, the fit statistics were χ^2^(250) = 708.29 (*p* < .00), AIC = 20496.56, BIC = 20561.94, RMSEA = .074 (90% CI = .067 - .080), CFI = .89, TLI = .87, SRMR = .06, and the output suggested that one more item was nonsignificant in this new model (item 19 on the indirect numeracy environment specific factor). This item was subsequently also dropped and this new model’s fit statistics were χ^2^(228) = 596.63(*p* < .00), AIC = 19672.66, BIC = 19735.42, RMSEA = .069 (90% CI = .062 - .076), CFI = .91, TLI = .89, SRMR = .06. After running this model, the output indicated that one more item was subsequently nonsignificant on the direct numeracy environment specific factor (item 43). After this run, the output did not indicate that any further modifications were needed, so the exploratory item dropping stopped and the model was chosen as the final model.

**Fig 1:**
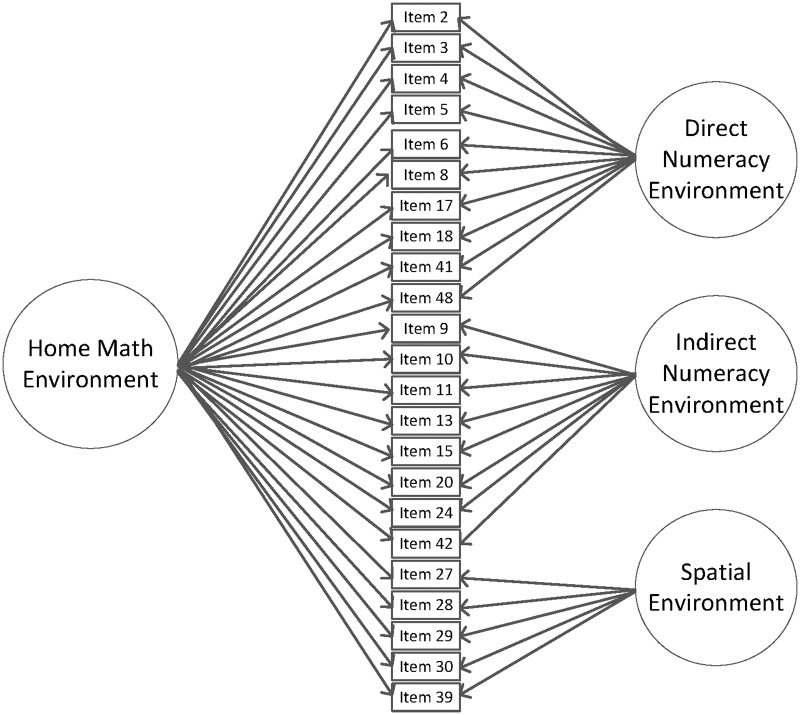
Final bifactor model, representing the general home math environment factor and three specific factors of direct numeracy environment, indirect numeracy environment, and spatial environment.

The final model included 23 items, 10 items on the direct numeracy environment specific factor, eight items on the indirect numeracy environment specific factor, and the remaining five items on the spatial environment specific factor. The model fit statistics were adequate, χ^2^(207) = 547.13 (*p* < .00), AIC = 18832.11, BIC = 18892.26, RMSEA = .070 (90% CI = .063 - .077), CFI = .91, TLI = .89, SRMR = .05. All factor loadings from this final model of the home math environment can be seen in Table 1 (see Fig 1 for final model).

### Relation of Home Math Environment to Parent Report of Child Skills

As a first step before moving onto the modeling specific to the second and third goals, a measurement model where all latent factors and measured variables were estimated and allowed to correlate was fitted. This allowed for a determination of the fit of all of the parts of the final model, before stepping through the directional specifications as dictated by the research questions. Modification indices indicated that the residuals of the Colorado Learning Disabilities Questionnaire (CLDQ) items on the child factor should be allowed to correlate. Additionally, modification indices indicated that residuals of certain items from the parent math anxiety factor should be allowed to correlate (item 1 with item 2, item 2 with item 4, item 3 with item 4, item 2 with item 5, item 3 with item 5, item 5 with item 6, and item 3 with item 6). The final model fit statistics for the measurement model after the residuals just listed were allowed to correlate were adequate χ^2^(627) = 1161.34 (*p* < .00), AIC = 31457.65, BIC = 31583.19, RMSEA = .050 (90% CI = .046 - .055), CFI = .91, TLI = .89, SRMR = .05.

Factor loadings of the latent factors formed in the measurement model (i.e. parent math anxiety and parent report of child skills) are displayed in Table 3. All factor loadings were adequate (i.e. greater than .20).

**Table 3:** Factor loadings and descriptive statistics for all measures used in the structural equation modeling.

Item	Factor loading	Mean	*SD*	Range	Skew
Child gender	—	1.55	.50	1–2	-.21
Child age	—	5.96	1.63	3.01–8.90	-.06
Parent gender	—	1.67	.47	1–2	-.73
Household Income	—	4.17	1.87	1–12	1.15
Parent ANS (*w*-score)	—	.31	.26	.10–2.42	4.76/1.48^a^
Parent math anxiety factor					
Looking through pages in a math book.	.69	1.87	1.22	1–5	1.16
Being asked to add up 976 and 777 in your head	.84	2.01	1.19	1–5	1.00
Determining the amount of change you should get back from a purchase involving several items.	.85	1.71	1.06	1–5	1.50
Calculating a tip at a restaurant without using a calculator	.79	1.67	1.05	1–5	1.51
Having someone explain bank interest rates as you decide on a savings account.	.67	2.10	1.23	1–5	.78
Being asked by a friend to answer the question: How long will it take to get to New York City if I drive 70 miles per hour?	.74	2.11	1.24	1–5	.80
Parent report of child skills factor					
Child math skill	.75	63.40	22.79	0–100	-.71
Child number skill	.87	74.63	20.39	1–100	-1.18
Child CLDQ math	.31	5.73	2.74	3–14	.83
Child spatial skill	.76	70.17	19.84	1–100	-.72
Child CLDQ spatial	.21	8.02	3.33	4–17	.58

*Note*. All factor loadings significant at *p* < .05.

*n* = 339, other than parent *w*-score, where *n* = 315, income, where *n* = 333, and parent gender where *n* = 338.

^a^Parent ANS skew is reported as before log-transformation/after log-transformation.

The correlations among the measures and latent factors of the measurement model are presented in Table 4. Results showed that the general home math environment factor was positively correlated with parent report of child skills (*r* = .23). After accounting for the general home math environment factor, the spatial environment specific factor was significantly negatively correlated with parent report of child skill (*r* = -.16), and the direct numeracy environment and indirect numeracy environment specific factors indicated only small and nonsignificant correlations with parent report of child skills. Other than child gender and parent gender, all other variables and latent factors were significantly correlated with the parent report of child skills factor.

**Table 4:** Correlations among all the components of the final structural equation model.

Variable	1.	2.	3.	4.	5.	6.	7.	8.	9.	10.	11.
1. Home math environment factor	1.00										
2. Direct numeracy environment factor	.00	1.00									
3. Indirect numeracy environment factor	.00	.00	1.00								
4. Spatial home environment factor	.00	.00	.00	1.00							
5. Household income	.08	-.03	.01	-.02	1.00						
6. Parent ANS	-.08	.08	.07	-.14*	.03	1.00					
7. Parent math anxiety factor	-.04	.04	-.11	.13	-.15*	-.14*	1.00				
8. Child gender	-.06	-.01	.02	-.02	-.02	-.02	-.05	1.00			
9. Child age	.07	-.45*	.31*	-.11	.12*	.01	-.12*	.02	1.00		
10. Parent gender	-.07*	.03	.06	-.06	-.06*	.00	.06*	.00	.03	1.00	
11. Parent report of child skills factor	.23*	-.06	.08	-.16*	.15*	.16*	-.16*	.03	.15*	.02	1.00

*Note*. In a bifactor model, the factors are set to be orthogonal.

* *p* < .05

**Fig 2:**
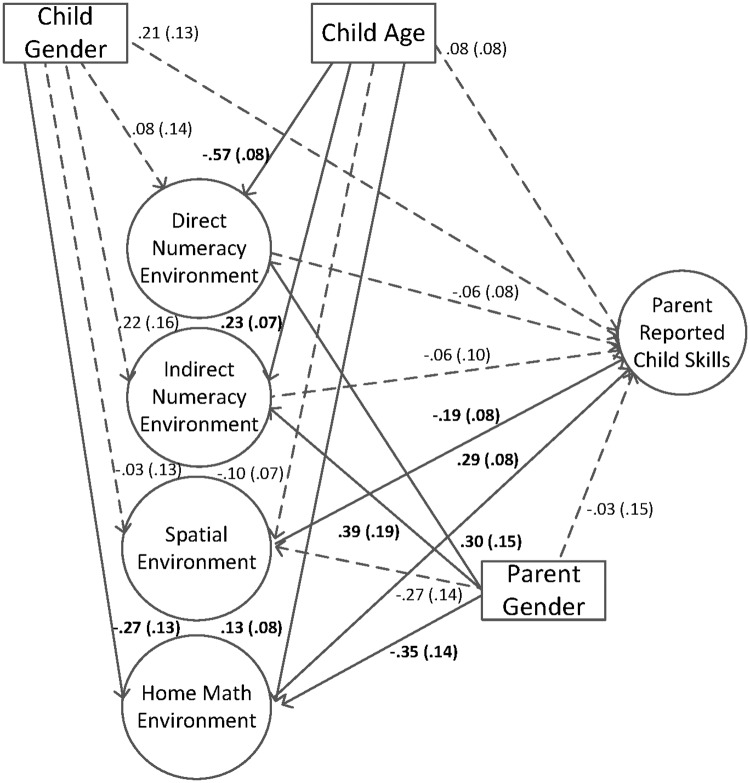
Home math environment factors predicting parent report of child skills. Standard errors are in parentheses. Solid lines and bolded text represent significant pathways, *p* < .05. Longdash lines represent non-significant pathways, *p* > .05.

After assessing the measurement model, we addressed the second goal by including the final bifactor model as part of a larger structural equation model testing the role of the home math environment in predicting parent report of child skills (see Fig 2). Child age, child gender and parent gender were included as covariates. The model fit statistics were adequate, χ^2^(391) = 827.19 (*p* < .00), AIC = 22976.91, BIC = 23059.58, RMSEA = .054 (90% CI = .052 - .063), CFI = .90, TLI = .88, SRMR = .05. The results indicated that the general home math environment factor positively predicted parent report of child skills (pathway = .29) and the spatial environment negatively predicted (pathway = -.19) parent report of child skills, when controlling for child gender, child age, and parent gender.

### Relation of Home Math Environment to Parent Report of Child Skills, Accounting for Alternative Explanations

**Fig 3:**
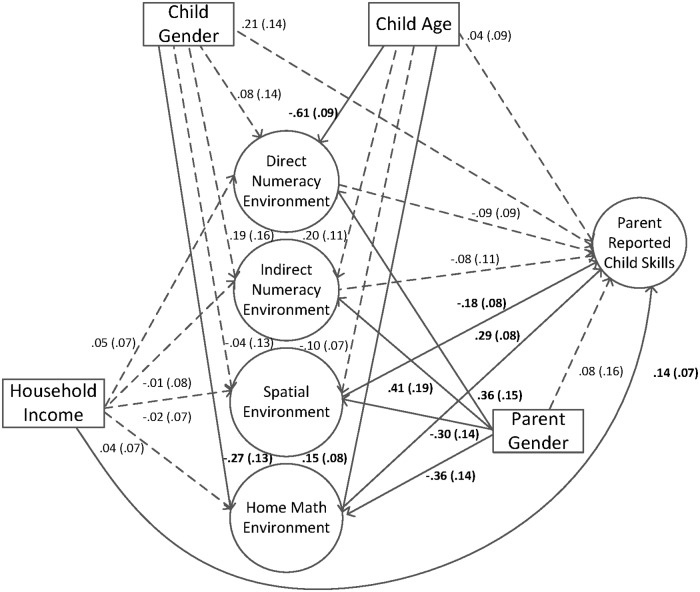
Home math environment factors and household income predicting parent report of child skills. Standard errors are in parentheses. Solid lines and bolded text represent significant pathways, *p* < .05. Longdash lines represent non-significant pathways, *p* > .05.

The third goal of this study involved a series of structural equation models where different alternative explanatory variables are added in one at a time, and then together, to examine the robustness of the prediction of the home math environment (HME) to parent report of child skills. In the first model, household income was added as a direct and indirect predictor of parent report of child skills along with the HME factors (see Fig 3). The model fit statistics were adequate, χ^2^(414) = 854.33 (*p* < .00), AIC = 22540.08, BIC = 22623.64, RMSEA = .057 (90% CI = .051 - .062), CFI = .90, TLI = .88, SRMR = .05. Results indicated that household income was not related to any of the HME factors, but it was positively directly related to parent report of child skills, in that higher household income was related to higher parent report of child skills (pathway = .14). In this model, the inclusion of income as a direct and indirect predictor of parent report of child skills did not affect the pattern of significance of the prediction of the HME factors to parent report of child skills (as seen in Fig 2).

**Fig 4:**
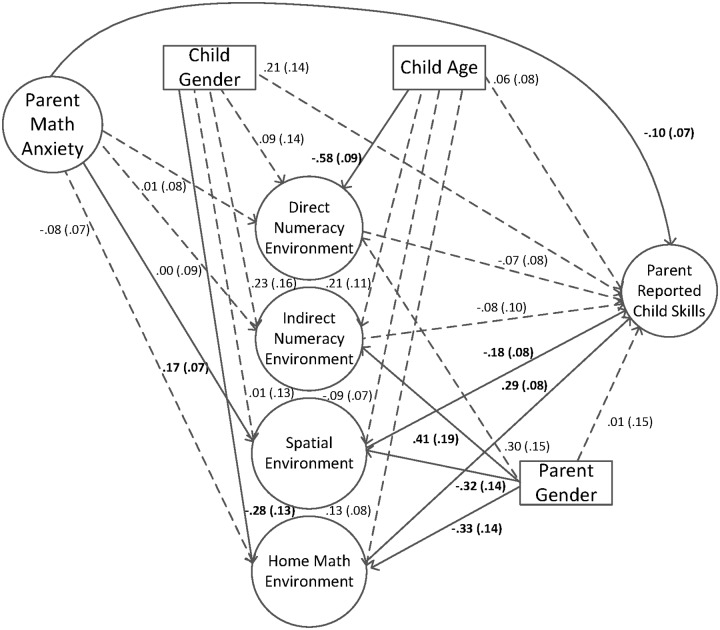
Home math environment factors and parent math anxiety predicting parent report of child skills. Standard errors are in parentheses. Solid lines and bolded text represent significant pathways, *p* < .05. Longdash lines represent non-significant pathways, *p* > .05.

For the second model of the third goal, parent math anxiety was added as a direct and indirect predictor of parent report of child skills along with the HME factors (see Fig 4). The model fit statistics were adequate, χ^2^(574) = 1082.48 (*p* < .00), AIC = 27635.89, BIC = 27738.08, RMSEA = .051 (90% CI = .047 - .056), CFI = .91, TLI = .90, SRMR = .06. Results indicated that parent math anxiety was not a significant direct predictor of parent report of child skills (pathway = -.10), but it was a significant indirect negative predictor of parent report of child skills through the spatial environment specific factor such that parents higher in math anxiety did more spatial activities with their children, which was negatively associated with child math skills. The inclusion of parent math anxiety into this model did not change the relations between the HME factors and parent report of child skills, in that the general home math environment factor was still a significant positive predictor of parent report of child skills and spatial environment specific factor was still a significant negative predictor of parent report of child skills, but the other HME factors were not.

**Fig 5:**
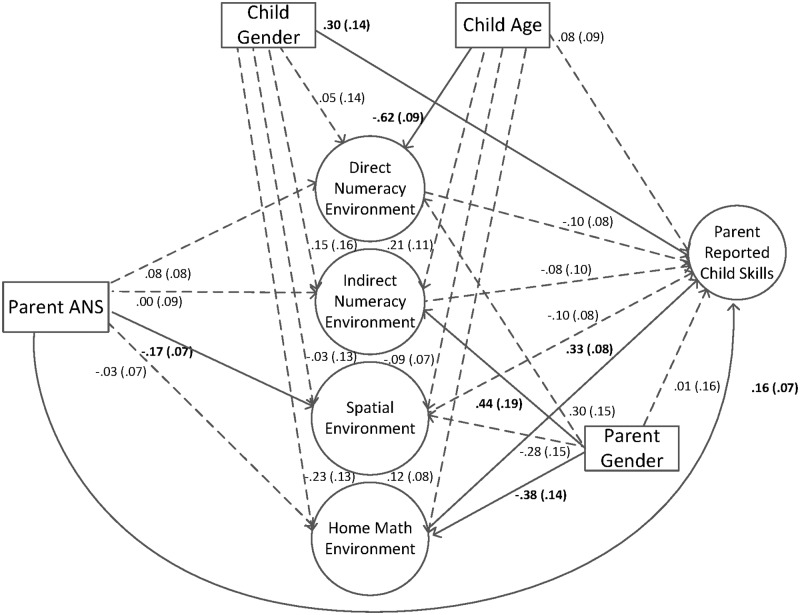
Home math environment factors and parent ANS predicting parent report of child skills. Standard errors are in parentheses. Solid lines and bolded text represent significant pathways, *p* < .05. Longdash lines represent non-significant pathways, *p* > .05.

In a third model, parent ANS was included as a direct and indirect predictor of parent report of child skills, along with the HME factors (see Fig 5). The model fit statistics were adequate, χ^2^(414) = 833.63 (*p* < .00), AIC = 21372.96, BIC = 21449.22, RMSEA = .057 (90% CI = .051 - .062), CFI = .90, TLI = .88, SRMR = .05. Results indicated that parent ANS has a positive, direct relation to parent report of child skills, in that parents with higher ANS had children with higher parent report of child skills (pathway = .16). Additionally, higher parent ANS was associated with lower scores on the spatial environment specific factor (pathway = -.17). In this model, including parent ANS into the model as a direct and indirect predictor of parent report of child skills, resulted in the spatial environment specific factor being a nonsignificant predictor of parent report of child skills, but the general home math environment factor was a significant positive predictor of parent report of child skills.

**Fig 6:**
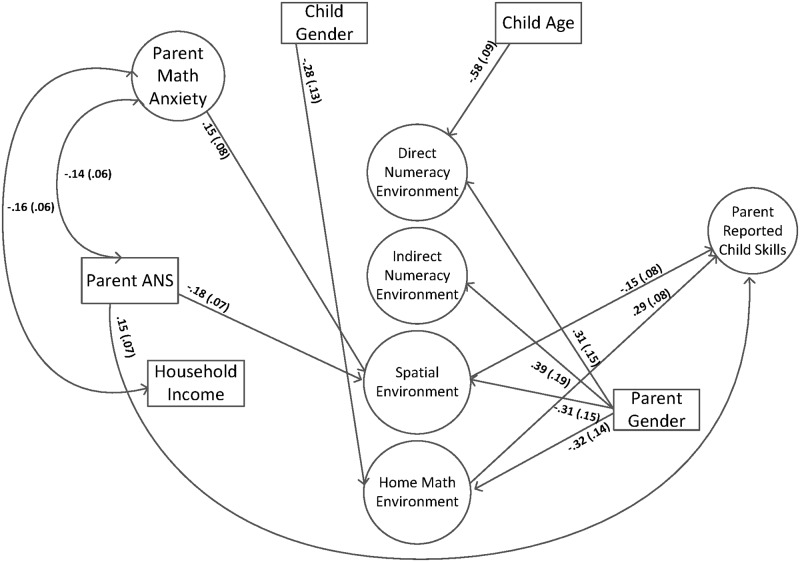
Home math environment factors, household income, parent math anxiety and parent ANS, predicting parent report of child skills. Standard errors are in parentheses. Solid lines and bolded text represent significant pathways, *p* < .05. Non-significant pathways are not displayed for simplicity.

In the fourth, and final model, household income, parent math anxiety and parent ANS were all included as a direct and indirect predictors of parent report of child skills, along with the HME factors (see Fig 6 for final significant pathways, and Table 5 for final non-significant results). The model fit the data adequately χ^2^(636) = 1184.62 (*p* < .00), AIC = 29469.05, BIC = 29582.31, RMSEA = .051 (90% CI = .046 - .055), CFI = .90, TLI = .89, SRMR = .06. The results of the model indicated that the general home math environment factor was a positive predictor of parent report of child skills (pathway = .29), and the spatial environment specific factor was a negative predictor of parent report of child skills (pathway = -.15). In terms of effect sizes, the general home math environment factor accounted for 8% of the variance in parent report of child skills, and the spatial environment specific factor accounting for 2% of the variance (note that *p* = .049 for this pathway). Additionally, there was a direct effect of parent ANS (pathway = .15, or 2% of the variance) on parent report of child skills, in that parents with a better ANS reported having children with higher skills. Parent ANS also had an indirect effect on parent report of child skills, through the spatial environment specific factor, in that parents with worse ANS scores reported participating in more spatially-related home activities which then predicted lower parent reported child math skills. This indirect effect accounted for 1% of the variance in parent report of child skills. Also, parent math anxiety was an indirect predictor of parent report of child skills, through the spatial environment specific factor, in that parents with higher math anxiety reported doing more spatially-related home activities (although, again, note that *p* = .046), which was then related to lower parent report of child skills. This indirect effect accounted for 1% of the variance in parent report of child skills.

**Table 5:** Path estimates and standard errors of the non-significant pathways from the final model.

Pathway	Estimate	Standard Error
Correlation of parent ANS with household income	.04	.06
Parent math anxiety predicting general home math environment factor	-.08	.07
Parent math anxiety predicting direct numeracy environment specific factor	.03	.08
Parent math anxiety predicting indirect numeracy environment specific factor	-.01	.09
Parent math anxiety predicting parent reported child skills	-.07	.07
Parent ANS predicting general home math environment factor	-.04	.07
Parent ANS predicting direct numeracy environment specific factor	.07	.08
Parent ANS predicting indirect numeracy environment specific factor	-.02	.10
Household income predicting general home math environment factor	.04	.07
Household income predicting direct numeracy environment specific factor	.04	.07
Household income predicting indirect numeracy environment specific factor	-.03	.08
Household income predicting spatial environment specific factor	.00	.07
Household income predicting parent reported child skills	.12	.07
Direct numeracy environment specific factor predicting parent reported child skills	-.08	.08
Indirect numeracy environment specific factor predicting parent reported child skills	-.07	.11
Parent gender predicting parent reported child skills	.04	.15
Child gender predicting direct numeracy environment specific factor	.10	.14
Child gender predicting indirect numeracy environment specific factor	.22	.17
Child gender predicting spatial environment specific factor	-.01	.13
Child gender predicting parent reported child skills	.23	.14
Child age predicting general home math environment factor	.12	.08
Child age predicting indirect numeracy environment specific factor	.22	.12
Child age predicting spatial environment specific factor	-.09	.07
Child age predicting parent reported child skills	.05	.09

Beyond these findings, the model indicated that the child’s gender was related to the general home math environment factor, in that parents with boys reported they did more home math activities than parents with girls. Also, child age predicted the direct numeracy environment specific factor, in that parents with younger children reported participating in more direct numeracy activities. The results for parent gender indicated that fathers reported doing more general home math environment activities as well as more spatial activities, and mothers reported doing more indirect and direct numeracy environment activities. Finally, parent math anxiety was negatively correlated with parent ANS and income.

## Discussion

### Factor Structure of the Home Math Environment

There is a growing literature concerning the role of the home learning environment, including both the home literacy environment and the home math environment (often called the “home numeracy environment”). This work includes proposing the importance of differentiating the “direct home environment” versus the “indirect home environment” (e.g., [11]; renamed to be the “formal numeracy” versus “informal numeracy” by [17]). The present analyses do support separating the home environment in this way, although the analyses also support adding in a new dimension to the home math environment, accounting for spatially-related activities (i.e. [18]) outside of the direct and indirect numeracy related activities. In total, the current data did support the direct and indirect numeracy environment model separation, as well as an additional spatial environment factor and, importantly, the data support that this model was best conceptualized when considering the residual variance for each of these specific factors after taking into account a general home math environment factor (i.e. the bifactor model).

The bifactor model is a latent variable approach, which allowed for all items from the home environment survey to load onto a general factor. This general factor represented the common variance across all the items, and therefore represents individual differences in the home math environment as measured by these items. Beyond this general factor, the bifactor structure specified three orthogonal specific factors. These specific factors represent factors that potentially explain item response variance not accounted for by the general factor. In this model, the direct and indirect numeracy environment factors and the spatial environment factor represented aspects of the home environment that were not accounted for by a broad home math environment definition, in other words, they represented the specific aspects proposed by LeFevre and colleagues (e.g., [11]) and Dearing and colleagues [18]. After accounting for the overall levels of the home learning environment related to math, items accounted for tasks that parents engage in with their children for the purpose of explicitly teaching quantitative skills (i.e. direct numeracy activities), other items accounted for real-world activities that incidentally teach quantitative skills (i.e. indirect numeracy activities), and finally other items accounted for activities that involve spatial reasoning. The activities associated with the spatial skills factor in the final model were all “indirect”, in that they described activities that were real-world in nature that also focused on spatial skills development. Interestingly, we found it difficult to construct items that were “direct” spatial activities, as these types of activities do not appear to commonly occur.

A bifactor model has not been considered previously in the literature when measuring the home math environment, and indeed we did not approach the present modeling with the bifactor model in mind. However, after seeing the high correlation (*r* > .80) between the factors in the planned two-factor models and the poor model fit of the single factor model, we decided to examine bifactor models. Bifactor models are becoming more common in psychology, where constructs are commonly best considered as a single common factor at the same time as having specific sub-factors [53]. In all cases, the bifactor model fit better than the accompanying correlated factor model, and better than the one factor model, lending support to the idea that the home math environment is captured best by this bifactor framework. A positive implication of using the bifactor model is that the factors are forced to be orthogonal to each other (by definition), there is no chance of highly correlated factors (such as the direct and indirect numeracy environment factors) causing a suppression effect (or other statistical artifacts) when modeled together predicting children’s skills. We suggest that other researchers consider the bifactor model in their own approaches to studying the home math environment.

### Relation between the HME and Parent Report of Child Skills

After settling on the final home math environment model, we then proceeded to determine the extent to which the home math environment predicted parent report of child skills. Results from this initial model indicated that the general HME factor positively predicted parent report of child skills, and the spatial environment specific factor negatively predicted parent report of child skills. Had we stopped here, we likely would have concluded that the general home math environment is important in predicting parent report of child skills, replicating previous work on the general learning environment, and more specifically the home math environment. However, we sought to test if this association of the home math environment with parent report of child skills persisted after including a set of known alternative explanatory variables.

### Relation of Home Math Environment to Parent Report of Child Skills, Accounting for Potential Alternative Explanations

We included household income, parent math anxiety and parent ANS one by one, and then simultaneously, into the model, conservatively testing the association of HME on parent report of child skills. We found that household income and parent math anxiety were not likely alternative explanations, as in both cases, the association of the general home math environment factor and the spatial environment specific factor with parent report of child skills remained significant when either were in the model. However, household income and parent math anxiety were direct predictors of parent report of child skills, in that higher household income was related to higher parent report of child skills, and higher parent math anxiety was related to lower parent report of child skills. Also, parent math anxiety was a significant predictor of the spatial environment specific factor, in that parents with higher math anxiety did more spatial activities, controlling for the general home math environment factor, which was then negatively associated with parent report of child skills. The reason for this finding is not clear, although it could be due to spatial activities being less obviously “math” related and therefore less anxiety-provoking for high math anxious parents.

Importantly, when parent ANS was added to the model, it was clear that this was an important alternative explanation to consider. The results showed that parent ANS directly predicted parent report of child skills, and did so at the expense of the negative spatial environment specific factor effect on parent report of child skills. Parent ANS performance was positively associated with parent report of child skills, and was related to fewer spatial environment activities, which was no longer associated with parent report of child skills. The focus of the present work was not to determine the underlying causes of the direct pathway between parent ANS and parent report of child skills. However, it is likely the case the direct pathway not only describes inherited genetic influences underlying math skills, but also environmental influences beyond the HME (as HME is already included in the model). For example, previous work has highlighted parent supportiveness, as an important mediator between parent math skills more broadly and child skills when also accounting for the home math environment [43]. We assume these possibilities underlie this pathway, and encourage more work to determine what more, past the HME, explains the direct association between parents and their children’s math skills as seen here when measured by parent report.

When all the possible alternative explanatory variables were measured together, it was found that the general home math environment factor remained as a significant positive predictor of parent report of child skills. Parents who reported doing more math-related activities in their home also reported that their children had higher math skills. This supports the literature indicating the importance of the home math environment on children’s math development, although interestingly seems to point to the global influence of the home math environment, rather than any specific aspect of the environment being the most important (e.g., just direct numeracy activities). This finding held up despite our inclusion of a host of alternative explanations including household income, parent math anxiety and parent ANS. It may be the case that there were other non-measured explanatory variables that would account for this association, including having the parents rate both the home activities and their children’s skills, but at the very least these results suggest that doing more activities in the home related to math skills is positively associated to parents reporting children with greater math skills.

In this final model, the negative prediction of the spatial environment to parent report child skills was significant in straight *p*-value cutoff terms, at *p* = .049, but it was a fairly weak relation, as it only explained 2% of the variance in parent report of child skills. This information, along with the knowledge that the relation was not significant in a simpler model that just included parent ANS, suggests this relation may be tentative. We therefore caution overly interpreting this finding. Both parent math anxiety and parent ANS continued to significantly predict the spatial environment factor, with the relations indicating that parents with poorer ANS performance and more math anxiety tended to do more spatial activities, after accounting for the general math environment. Given there is a finite amount of time to spend with children doing math activities in the home, we believe this suggests that parents who have weaker math skills and/or more math anxiety tend to choose to do activities that are not numeracy related when participating in home math environment activities.

Household income and parent math anxiety, as well as parent ANS and parent math anxiety, were significantly correlated with each other in the expected directions, and after including all these possible alternative explanatory variables, only parent ANS remained as a direct predictor of parent report of child skills. Unfortunately, parent skills typically have not been considered in previous models of the home math environment, and we think it should more regularly be included. In a recent article setting out to develop a home numeracy model, only parent attitudes towards numeracy and academic expectations were considered, and not the parent’s own numeracy skills [17]. We acknowledge we did not consider parent attitudes (other than parent math anxiety) or expectations in the current analyses, and therefore we cannot say how these variables would have changed our models, if at all. The only previous study that did include parent skills, found that maternal spatial skills directly predicted their daughters’ numerosity and spatial performance, but did not predict the general home learning environment outside of included socioeconomic predictors [18].

### Limitations

Like any study, there are limitations to consider. First, there are multiple limitations when considering the measures. Response bias is a concern, in that social desirability may have resulted in more parents indicating higher frequencies for each home math environment activity than was true. However, given that a few items were rated by parents as being done very infrequently, this may not be a large concern. Also, the online data collection allowed for a large sample to be collected, but resulted in limited assessment possibilities for parent and child math skills. Parent math skill was limited to a measure of numerosity, and child math ability was limited to only parent report. A recent report found that if parents reported at all (as parents reported “unsure” for 0% to 40% of the math skills asked), they were able to relatively accurately rate their children’s math skills (across a wide range of math skills); and if incorrect, parents tend to overestimate their children’s math skills [54]. Interestingly, although parents’ reports of their children’s scores were relatively accurate to slightly overestimated at the mean level, the reported standard deviations of the actual performance measures versus parent reports were quite similar [54]. Despite this, self-report methods are likely not as accurate as standardized measures given by a trained administrator, which would have been more ideal. Additionally, it would have taken away the possibility of common-rater variance accounting for the relations we see here. We also acknowledge that parent ANS performance is not an ideal variable to represent parent math skills more broadly, given the uncertain relation of ANS to adult advanced math performance [55]. As such, our finding should be considered within the broader literature.

Another limitation is we could only measure one parent, and therefore have a limited view of the home environment. The results indicating that parent gender was associated with differing types of participation in the home math environment activities warrants further analyses. Also, when controlling for internal parent factors (i.e. parent math anxiety and parent skills), it is important to consider both parents. For example, it could be the case that one parent has higher math anxiety than the other, and the parent with less math anxiety provides a larger proportion of the activities considered as part of the direct numeracy environment. We were not able to consider such nuances.

In order to get a large enough sample, we made the decision to use a wider child age range than typical for the literature examining the home math environment. It could be the case that home math environment is different for younger children versus school-aged children. To check this, we did secondary analyses that split the current sample into children less than 6yrs old (preschool and kindergarten in the U.S.), and children 6yrs old and older, which represented an almost exact median split. We found that the best fitting home math environment model for both age groups was the same model we selected using the full sample, although, as can be expected, slightly different items were kept for each factor (results available in the Supporting Information, S2 File). We feel confident that the broader conclusion of the bifactor model with the three specific factors of direct numeracy environment, indirect numeracy environment, and the spatial environment, represents the activities occurring in homes with a wide-range of children, but likely different activities are done in homes with children of different ages. It may also be the case that the total frequency of activities decreases as children get older, a question we do not directly test here.

The measurement model indicated that other components in the model would be related to the parent report of child skills factor, but when all the effects were modeled simultaneously, it is possible that multicollinearity played an effect in reducing the influence of certain components of the model, specifically in the final model with all variables modeled simultaneously. Finally, it would have been ideal to take a developmental approach when considering these effects. Even more importantly, the best way (and could be argued the only way) to control for gene-environment processes is to use genetically-sensitive designs, such as extended twin designs. However, these data are not available presently, and this is an avenue for future research.

Finally, we purposely included more items than necessary for the HME measure in our data collection, as we intended to reduce the items to create the best possible model of the HME. To do this, we took a model fitting approach which resulted in just over 50% of the items being dropped in the final factor model. We suggest that future researchers use our final factor model of HME as a base for HME assessment, and increase the utility of it through more item testing. For example, more items might be created for the spatial environment specific factor, potentially with the ability to break out an indirect and direct spatial environment, which will likely increase the precision of this factor (and the others).

### Conclusions

In general, we replicated the previous work highlighting the importance of separating the home math environment into direct, indirect, and spatial components. We did so while also highlighting the importance of defining the general home math environment, before separating the environment into specific components. The association of the general home math environment factor, and, to a lesser extent, the spatial environment specific factor, with parent report of child skills remained even after accounting for various other explanatory variables. We encourage others working to understand the home learning environment to consider that parents provide both their genes and the home learning environment related to their children’s performance, and these competing influences need to be accounted for when considering the role of the home math environment.

## Supporting Information

S1 File Blank Questionnaire file. (DOCX)

S2 File Results from splitting ages into younger and older samples. (DOCX)

S1 Data Final complete raw data. (XLSX)

S2 Data Mplus dat file for HNE factor modeling. (DAT)

S3 Data Mplus dat file for SEM modeling. (DAT)

S1 Code SAS code used to create S1 Data and run analyses presented in paper. (SAS)

S2 Code Mplus code: HNE factor model_one factor. (TXT)

S3 Code Mplus code: HNE factor model_two factor_directvsindirect. (TXT)

S4 Code Mplus code: HNE factor model_two factor_HSEvsHNEN. (TXT)

S5 Code Mplus code: HNE factor model_three factors. (TXT)

S6 Code Mplus code: HNE factor model_four factors. (TXT)

S7 Code Mplus code: HNE factor model_bifactor_directvsindirect. (TXT)

S8 Code Mplus code: HNE factor model_bifactor_HSEvsHNE. (TXT)

S9 Code Mplus code: HNE factor model_bifactor_three factors. (TXT)

S10 Code Mplus code: HNE factor model_bifactor_three factors(reducedv1). (TXT)

S11 Code Mplus code: HNE factor model_bifactor_three factors(reducedv2). (TXT)

S12 Code Mplus code: HNE factor model_bifactor_three factors(reducedv3). (TXT)

S13 Code Mplus code: Correlatedfactorsmodel_3factormodel. (TXT)

S14 Code Mplus code: SEM_threefactors _HNEparentgender. (TXT)

S15 Code Mplus code: SEM_threefactors_ HNEincome. (TXT)

S16 Code Mplus code: SEM_threefactors_HNEmathanxiety. (TXT)

S17 Code Mplus code: SEM_threefactors_ HNEparentW. (TXT)

S18 Code Mplus code: SEM_threefactors_ fullfinalmodel. (TXT)
