# Supplementary material for: Understanding the Home Math Environment and Its Role in Predicting Parent Report of Children’s Math Skills
Source: PLoS One. 2016 Dec 22;11(12):e0168227. doi: 10.1371/journal.pone.0168227 (PMC5179117; doi:10.1371/journal.pone.0168227)
Supplement: S1 File — (DOCX) [file pone.0168227.s001.docx]

1. How many children do you have? ___________
2. Tell us about your child who is between the ages of 3 to 8 years old. If you have more than one child between the ages of 3 and 8, please pick one of your children in this age range as the focus for your answers throughout this entire survey. All answers should be about the same child
   1. Date of Birth (dd/mm/yyyy) ☐ boy ☐girl

(for parents of younger age range) Compared to 100 people this child’s age, he/she would be better than ________ of them with numbers, such as counting or know number names

Compared to 100 people this child’s age, he/she would be better than ________ of them with math

Compared to 100 people this child’s age, he/she would be better than ________ of them with science

Compared to 100 people this child’s age, he/she would be better than ________ of them with writing

Compared to 100 people this child’s age, he/she would be better than ________ of them with reading

Compared to 100 people this child’s age, he/she would be better than ________ of them with spatial skills, such as doing a puzzle

**Please read each statement and decide how well it describes your child. Mark your answer by circling the appropriate number. Please do not leave any statement unmarked.**

|  | **Never/**  **not at all** | **Rarely/**  **a little** | **Sometimes** | **Frequently/**  **quite a lot** | **Always/**  **a great deal** |
| --- | --- | --- | --- | --- | --- |
| 1. Does/did your child have difficulty with spelling? | **1** | **2** | **3** | **4** | **5** |
| 1. Does/did your child have difficulty learning letter names? | **1** | **2** | **3** | **4** | **5** |
| 1. Does/did your child have difficulty learning phonics (sounding out words)? | **1** | **2** | **3** | **4** | **5** |
| 1. Does/did your child read slowly? | **1** | **2** | **3** | **4** | **5** |
| 1. Does/did your child read below grade or expectancy level? | **1** | **2** | **3** | **4** | **5** |
| 1. Does/did your child require extra help in school because of problems in reading and spelling? | **1** | **2** | **3** | **4** | **5** |
| 1. Does/did your child have poor understanding of interpersonal space? | **1** | **2** | **3** | **4** | **5** |
| 1. Does/did your child have difficulty knowing how others are reacting? | **1** | **2** | **3** | **4** | **5** |
| 1. Does/did your child have trouble understanding how others are feeling? | **1** | **2** | **3** | **4** | **5** |
| 1. Does/did your child make comments that show a lack of understanding of social situations, such as inappropriate jokes or insensitive remarks? | **1** | **2** | **3** | **4** | **5** |
| 1. Does/did your child have difficulty making or keeping friends? | **1** | **2** | **3** | **4** | **5** |
| 1. Does/did your child isolate (him/her)self in social situations? | **1** | **2** | **3** | **4** | **5** |
| 1. Does/did your child feel anxious or out-of-place in new social situations? | **1** | **2** | **3** | **4** | **5** |
| 1. Is/was your child’s handwriting spatially disorganized? | **1** | **2** | **3** | **4** | **5** |
| 1. Do/did your child’s papers look disorganized or messy? | **1** | **2** | **3** | **4** | **5** |
| 1. On arithmetic problems, does/did your child have difficulty keeping the numbers lined up in columns? | **1** | **2** | **3** | **4** | **5** |
| 1. Do/did your child’s drawings look immature for his/her age? | **1** | **2** | **3** | **4** | **5** |
| 1. Was/is your child worse at math than at reading and spelling? | **1** | **2** | **3** | **4** | **5** |
| 1. Does/did your child make careless errors in math, such as adding when the sign indicates subtraction? | **1** | **2** | **3** | **4** | **5** |
| 1. Does/did your child have trouble learning new math concepts such as carrying or borrowing? | **1** | **2** | **3** | **4** | **5** |

1. How important is it that your child to do well in math?

Not important Very important

At all

1 2 3 4 5 6 7 N/A

1. How upset would you be if your child got a low grade or evaluation in math?

Not at all

Upset Very upset

1 2 3 4 5 6 7 N/A

1. I feel that it is important for my child to do better in math than other

children in their classroom.

Strongly Strongly

disagree agree

1 2 3 4 5 6 7 N/A

1. To do well in math, my child has to try

A little A lot

1 2 3 4 5 6 7 N/A

1. If your child worked harder at math, how much would his or her performance

in math change?

A little A lot

1 2 3 4 5 6 7 N/A

1. How often do you let your child know what you think about his or her math ability?

rarely very often

1 2 3 4 5 6 7 N/A

1. How much do you think your opinion about your child’s math ability matter to your

child?

A little A lot

1 2 3 4 5 6 7 N/A

1. How much can you do to insure that your child achieve at a high level in

math?

A little A lot

1 2 3 4 5 6 7

11. Is it important for my child to be read to every day?

Not important Very Important

1 2 3 4 5

12. Is it important for my child to be exposed to math concepts every day?

Not important Very Important

1 2 3 4 5

1. What was your overall (unweighted) high school GPA?

☐0-1.0 ☐1.1-2.0 ☐2.1-3.0 ☐3.1-3.5 ☐3.6-4.0

b) If you remember, what was your exact overall GPA ? _________

1. If you are attending or have attended college, what is/was your overall GPA?

☐0-1.0 ☐1.1-2.0 ☐2.1-3.0 ☐3.1-3.5 ☐3.6-4.0 ☐4.1-4.5

b) If you remember, what was your exact overall GPA? _________

1. What was your overall unweighted college GPA maximum possible score?
   1. 4.0
   2. 4.5
   3. Other __________
2. Compared to 100 people my age, I would be better than ________ of them with **computers**
3. Compared to 100 people my age, I would be better than ________ of them with **math**
4. Compared to 100 people my age, I would be better than ________ of them with **science**
5. Compared to 100 people my age, I would be better than ________ of them with **writing**
6. Compared to 100 people my age, I would be better than ________ of them with **reading**
7. Compared to 100 people my age, I would be better than ________ of them with **spatial** skills, like reading maps or doing puzzles
8. The SAT (also previously known as the Standard Aptitude Test and Scholastic Achievement Test) and the ACT are standardized tests commonly taken by high school students for admission to colleges and universities in the United States.

Did you take either the SAT or ACT (if you took both, please select the one below that best remember your scores for?

___SAT

___ACT

___Neither

(if selected SAT)

1. What was your overall SAT score? _________
2. What was the maximum possible score for the SAT when you took it?
   1. 1600
   2. 2400
   3. Other _________
3. What was your score on the Critical Reading portion of the SAT (also known as the Verbal section)? If you are not certain, please make your best guess. If you do not remember at all or did not take this portion of the test, please leave this field blank.

____ of 800 (leave blank if unknown or not applicable)

a. How confident are you that your Critical Reading score estimate is accurate? _________ (not at all 0 to very 100)

1. What was your score on the Mathematics portion of the SAT (also known as the Quantitative or Calculation section)? If you are not certain, please make your best guess. If you do not remember at all or did not take this portion of the test, please leave this field blank.

____ of 800 (leave blank if unknown or not applicable)

a. How confident are you that your Mathematics score estimate is accurate?

_________ (not at all 0 to very 100)

1. What was your score on the Writing portion of the SAT?*

**Please note this section was not officially added to the SAT until March 2005 and those who took the test before March 2005 likely do not have scores for this test.*

If you took this portion of the test, but are not certain of your scores, please make your best guess. If you do not remember your scores at all or did not take this portion of the test, please leave this field blank.

_______ of 800 (leave blank if unknown or not applicable)

a. How confident are you that your Writing score estimate is accurate?

_________ (not at all 0 to very 100)

(if selected ACT)

1. What was your ACT Composite score?

If you are not certain, please make your best guess. If you do not remember at all, please leave this field blank.

____ of 36 (leave blank if unknown or not applicable)

a) How confident are you that your Composite score estimate is accurate?

_________ (not at all 0 to very 100)

1. What was your score on the English portion of the ACT?

If you are not certain, please make your best guess. If you do not remember at all or did not take this portion of the test, please leave this field blank.

____ of 36 (leave blank if unknown or not applicable)

a) How confident are you that your English score estimate is accurate?

_________ (not at all 0 to very 100)

1. What was your score on the Mathematics portion of the ACT?

If you are not certain, please make your best guess. If you do not remember at all or did not take this portion of the test, please leave this field blank.

____ of 36 (leave blank if unknown or not applicable)

a. How confident are you that your Mathematics score estimate is accurate?

_________ (not at all 0 to very 100)

1. What was your score on the Reading portion of the ACT?

If you are not certain, please make your best guess. If you do not remember at all or did not take this portion of the test, please leave this field blank.

____ of 36 (leave blank if unknown or not applicable)

a. How confident are you that your Reading score estimate is accurate?

_________ (not at all 0 to very 100)

1. What was your score on the Science portion of the ACT?

If you are not certain, please make your best guess. If you do not remember at all or did not take this portion of the test, please leave this field blank.

____ of 36 (leave blank if unknown or not applicable)

a. How confident are you that your Science score estimate is accurate?

_________ (not at all 0 to very 100)

1. What was your score on the Writing portion of the ACT?

If you are not certain, please make your best guess. If you do not remember at all or did not take this portion of the test, please leave this field blank.

____ of 36 (leave blank if unknown or not applicable)

1. How confident are you that your Writing score estimate is accurate?

_________ (not at all 0 to very 100)

Some individuals feel anxiety when in certain situations involving mathematics. Please rate your level of anxiety when considering the following situations:

|  | Low anxiety | Some anxiety | Moderate anxiety | Quite a bit of anxiety | High anxiety |
| --- | --- | --- | --- | --- | --- |
| 1. Looking through pages in a math book. | 1 | 2 | 3 | 4 | 5 |
| 1. Being asked to add up 976 and 777 in your head | 1 | 2 | 3 | 4 | 5 |
| 1. Determining the amount of change you should get back from a purchase involving several items. | 1 | 2 | 3 | 4 | 5 |
| 4) Calculating a tip at a restaurant without using a calculator | 1 | 2 | 3 | 4 | 5 |
| 5) Having someone explain bank interest rates as you decide on a savings account. | 1 | 2 | 3 | 4 | 5 |
| 6) Being asked by a friend to answer the question: How long will it take to get to New York City if I drive 70 miles per hour? | 1 | 2 | 3 | 4 | 5 |

The following questions ask for your opinion about the role of the family and the school in educating children. Please circle the number that indicates the extent to which you generally agree with each statement

|  | Strongly Disagree | Disagree | No opinion | Agree | Strongly Agree |
| --- | --- | --- | --- | --- | --- |
| 1. Once a child is in school, the school has the main responsibility for his/her education. | 1 | 2 | 3 | 4 | 5 |
| b) Parents have the most influence on the development of the child's attitudes and beliefs. | 1 | 2 | 3 | 4 | 5 |
| c) Teachers at school should play a bigger part in the child's development than the parents. | 1 | 2 | 3 | 4 | 5 |
| d) Parents should continue to teach their child, even after the child enters school. | 1 | 2 | 3 | 4 | 5 |
| e) A child's success at school depends on how much his/her parents teach him/her at home | 1 | 2 | 3 | 4 | 5 |

In your opinion, how important is it for children to reach the following benchmarks prior to entering kindergarten?

|  | Not Important |  |  |  | Very Important |
| --- | --- | --- | --- | --- | --- |
| Count to 10 | 0 | 1 | 2 | 3 | 4 |
| Count to 100 | 0 | 1 | 2 | 3 | 4 |
| Identify/recognize written numbers | 0 | 1 | 2 | 3 | 4 |
| Simple sums | 0 | 1 | 2 | 3 | 4 |
| Solve basic word problems (e.g., Two cats are on a porch and two more joined them. How many cats are on the porch now?) for sums less than 8 | 0 | 1 | 2 | 3 | 4 |
| Use the terms “more than” and “less than” | 0 | 1 | 2 | 3 | 4 |
| Accurately count 1 to 15 objects in a row | 0 | 1 | 2 | 3 | 4 |
| Count or put out 1, 2, 3, 4, or 5 objects from a larger group of objects | 0 | 1 | 2 | 3 | 4 |
| Rehearse the alphabet | 0 | 1 | 2 | 3 | 4 |
| Identify/recognize alphabet letters | 0 | 1 | 2 | 3 | 4 |
| Print name | 0 | 1 | 2 | 3 | 4 |
| Print alphabet letters | 0 | 1 | 2 | 3 | 4 |
| Sound out three letter words |  |  |  |  |  |

**About how many children’s books do you have in your home?**

| None | 1 - 5 | 6 - 10 | 11 - 20 | 21 - 30 | 31 - 50 | 51 - 75 | 65 - 100 | 101 - 150 | More than 150 |
| --- | --- | --- | --- | --- | --- | --- | --- | --- | --- |

**About how many children’s books *that involve math in some way* do you have in your home?**

| None | 1 - 5 | 6 - 10 | 11 - 20 | 21 - 30 | 31 - 50 | 51 - 75 | 65 - 100 | 101 - 150 | More than 150 |
| --- | --- | --- | --- | --- | --- | --- | --- | --- | --- |

**About how many adult’s books do you have in your home?**

| None | 1 - 5 | 6 - 10 | 11 - 20 | 21 – 30 | 31 - 50 | 51 - 75 | 65 - 100 | 101 - 150 | More than 150 |
| --- | --- | --- | --- | --- | --- | --- | --- | --- | --- |

Please list the names of any children’s books you read with your child(ren), that you feel are related to number or math learning.

________________________________________________________________________________________________________________________________________________

Please list any toys and/or games you think are good for number or math learning

________________________________________________________________________________________________________________________________________________

ON AVERAGE, how often do you do each of the following with your child outside of school? We are trying to determine what children and their parents do inside the home that might be related to children’s math achievement in school. If you do something in your home that you believe may be related to math learning, but it is not listed, please list it in the “Other” fields below.

For each activity below, please select the option that indicates how often you did each.

|  | Never | Monthly or less | Less than once a week, but a few times a month (1-3 times) | About once a week | A few times a week (2-4 times) | Almost daily |
| --- | --- | --- | --- | --- | --- | --- |
| Use number or arithmetic flashcards | 0 | 1 | 2 | 3 | 4 | 5 |
| Identify names of written numbers | 0 | 1 | 2 | 3 | 4 | 5 |
| Play with numerical magnets | 0 | 1 | 2 | 3 | 4 | 5 |
| Counting objects | 0 | 1 | 2 | 3 | 4 | 5 |
| Sort things by color, shape, or size | 0 | 1 | 2 | 3 | 4 | 5 |
| Count down (10, 9, 8, 7…) | 0 | 1 | 2 | 3 | 4 | 5 |
| Learning simple sums (i.e., 2+2 = 4) | 0 | 1 | 2 | 3 | 4 | 5 |
| Printing numbers | 0 | 1 | 2 | 3 | 4 | 5 |
| Talk about money when shopping (e.g., “which costs more?”) | 0 | 1 | 2 | 3 | 4 | 5 |
| Measure ingredients when cooking | 0 | 1 | 2 | 3 | 4 | 5 |
| Bring timed | 0 | 1 | 2 | 3 | 4 | 5 |
| Playing with calculators | 0 | 1 | 2 | 3 | 4 | 5 |
| Making collections | 0 | 1 | 2 | 3 | 4 | 5 |
| “Connect-the-dots” activities | 0 | 1 | 2 | 3 | 4 | 5 |
| Using calendars and dates | 0 | 1 | 2 | 3 | 4 | 5 |
| Have your child wear a watch | 0 | 1 | 2 | 3 | 4 | 5 |
| Use number activity books | 0 | 1 | 2 | 3 | 4 | 5 |
| Read number storybooks | 0 | 1 | 2 | 3 | 4 | 5 |
| Play board games with a die or spiner | 0 | 1 | 2 | 3 | 4 | 5 |
| Play card games | 0 | 1 | 2 | 3 | 4 | 5 |
| Identifying sounds of alphabet letters |  |  |  |  |  |  |
| Printing letters |  |  |  |  |  |  |
| Use computer or video games to do drawing or painting or matching and playing with shapes | 0 | 1 | 2 | 3 | 4 | 5 |
| Uses a computer or video games to do addition, subtraction, or other math activities | 0 | 1 | 2 | 3 | 4 | 5 |
| Uses a computer or video games to do spatial tasks (such as the game Tetris) | 0 | 1 | 2 | 3 | 4 | 5 |
| Interact with clocks (such as pointing out to your child where the big hand and the little hand on the clock are and discussing what time it must be) | 0 | 1 | 2 | 3 | 4 | 5 |
| Count out money | 0 | 1 | 2 | 3 | 4 | 5 |
| Play with puzzles (such as picture puzzles, tangrams, slide puzzles, 3D puzzles) | 0 | 1 | 2 | 3 | 4 | 5 |
| Draw maps (such as treasure hunt maps) | 0 | 1 | 2 | 3 | 4 | 5 |
| Draw plans for houses, forts, castles, or other buildings or layouts | 0 | 1 | 2 | 3 | 4 | 5 |
| Measure the length and width of things | 0 | 1 | 2 | 3 | 4 | 5 |
| Use kits to build models (such as airplanes, animals, dinosaurs, doll houses) | 0 | 1 | 2 | 3 | 4 | 5 |
| Guess the number of things (such as candies in a jar) | 0 | 1 | 2 | 3 | 4 | 5 |
| Add or subtract numbers in your head with your child | 0 | 1 | 2 | 3 | 4 | 5 |
| Compare the sizes of numbers (such as 5 is more than 4) | 0 | 1 | 2 | 3 | 4 | 5 |
| Play with Legos or other building blocks | 0 | 1 | 2 | 3 | 4 | 5 |
| Play with an abacus | 0 | 1 | 2 | 3 | 4 | 5 |
| Keeping track of money with a Pig-E-Bank | 0 | 1 | 2 | 3 | 4 | 5 |
| Play with dominos | 0 | 1 | 2 | 3 | 4 | 5 |
| Fold or cut paper to make 3D objects (such as origami, paper planes) | 0 | 1 | 2 | 3 | 4 | 5 |
| Use scales | 0 | 1 | 2 | 3 | 4 | 5 |
| Play with a math mat | 0 | 1 | 2 | 3 | 4 | 5 |
| Note numbers on signs when driving or walking with children | 0 | 1 | 2 | 3 | 4 | 5 |
| Use numbers when referring to temperatures, time, and dates | 0 | 1 | 2 | 3 | 4 | 5 |
| Learn and sing math songs (such as Schoolhouse Rock) | 0 | 1 | 2 | 3 | 4 | 5 |
| Do math word problems | 0 | 1 | 2 | 3 | 4 | 5 |
| Games in the car that involve counting and/or math | 0 | 1 | 2 | 3 | 4 | 5 |
| Helping with math homework | 0 | 1 | 2 | 3 | 4 | 5 |
| Doing math in reference to sports (calculating batting averages, etc) | 0 | 1 | 2 | 3 | 4 | 5 |
| Recite numbers in order |  |  |  |  |  |  |
| Read any storybooks |  |  |  |  |  |  |
| Identify names of written alphabet letters |  |  |  |  |  |  |
| Recite numbers in order |  |  |  |  |  |  |
| Other: | 0 | 1 | 2 | 3 | 4 | 5 |
| Other: | 0 | 1 | 2 | 3 | 4 | 5 |
| Other: | 0 | 1 | 2 | 3 | 4 | 5 |
| Other: | 0 | 1 | 2 | 3 | 4 | 5 |
| Other: | 0 | 1 | 2 | 3 | 4 | 5 |

Dots Task

(Instructions)

Below is a link that you can click that will bring you to a task that measures your approximate sense of numbers. This is on an external website. Please read these instructions before clicking on the link.

After you click the link you will get instructions and practice and then complete the assessment. When you are finished, it will take a moment to compile your results. Then click "View Your Results" and it will bring you to an online PDF file. Please copy and paste the link to the PDF file in the field below. You are welcome to also save this to your computer if you are interested in the results.

Click here to go to the task (<http://panamath.org/expt5_fsu/>)

Please paste the link of the pdf document that is created for you here:

Please write the test number here:

1. Which of the following best describes the area that you currently live in?
   1. Urban or large city
   2. Suburban or small city
   3. Rural
2. What is your gender?
   1. Female
   2. Male
   3. Prefer not to answer
3. How old are you? __________
4. What is your ethnicity? (check one):
   1. ___ Not Hispanic or Latino
   2. ___ Hispanic or Latino
   3. ___ would prefer to not answer
5. What is your race? (check one):

___ American Indian or Alaska Native

___ Asian

___ Native Hawaiian or Other Pacific Islander

___ Black or African American

___ White

___ Other (please specify: )

___ Mixed (more than one of the above)

___ would prefer to not answer

1. What is your *current* household income? (check one)

___ less than $10,000 ___130,000 – 149,000

___ $10,000 – 29,000 ___150,000 – 169,000

___ $30,000 – 49,000 ___170,000 – 189,000

___ $50,000 – 69,000 ___190,000 – 209,000

___ $70,000 – 89,000 ___210,000 or more

___ $90,000 – 109,000 ___ Don’t know

___110,000 – 129,000 ___ would prefer to not answer

1. What is your level of school completed? (check one)

___ Grade 6 or less

___ Grade 7 – 12 (without graduating high school or equivalent)

___ Graduated high school or high school equivalent

___ Some college

___ Graduated from 2-year college

___Graduated from 4-year college

___ Attended graduate or professional school without graduating

___ Completed graduate or professional school

___ Would prefer to not answer

1. Which of the following categories best describes your primary occupation?
   1. Homemaker
   2. Student
   3. Unemployed
   4. Retired
   5. Day laborer; janitor; house cleaner; farm worker; food counter sales; food preparation worker; busboy
   6. Garbage collector; short-order cook; cab driver; shoe sales; assembly line workers; masons; baggage porter
   7. Painter; skilled construction trade; sales clerk; truck driver; cook; sales counter or general office clerk
   8. Automobile mechanic; typist; locksmith; farmer; carpenter; receptionist; construction laborer; hairdresser
   9. Machinist; musician; bookkeeper; secretary; insurance sales; cabinet maker; personnel specialist; welder
   10. Supervisor; librarian; aircraft mechanic; artist or artisan; electrician; administrator; military enlister personnel; buyer
   11. Nurse; skilled technician; medical technician; counselor; manager; police or fire personnel; financial manager; physical, occupational, speech therapist
   12. Mechanical, nuclear, or electrical engineer; educational administrator; veterinarian; military officer; elementary, high school, or special education teacher
   13. Physician; attorney; professor; chemical or aerospace engineer; judge; CEO; senior manager; public official; psychologist; pharmacist; accountant
   14. Other (please specify): __________
   15. would prefer to not answer
2. **Please describe your job or occupation (if any) in more detail (e.g., what field is the job in, what is your job title) _____________________**

Please write down any questions, comments, or issues that you may have had with this questionnaire.

____________________________________________________________
