# Supplementary material for: Understanding the Home Math Environment and Its Role in Predicting Parent Report of Children’s Math Skills
Source: PLoS One. 2016 Dec 22;11(12):e0168227. doi: 10.1371/journal.pone.0168227 (PMC5179117; doi:10.1371/journal.pone.0168227)
Supplement: S2 File — (DOCX) [file pone.0168227.s002.docx]

| Model | *χ²* | *df* | *p* | AIC | Adj BIC | RMSEA | RMSEA lower bound | RMSEA upper bound | CFI | TLI | SRMR |
| --- | --- | --- | --- | --- | --- | --- | --- | --- | --- | --- | --- |
| 1 Factor: Home Environment | 2375.31 | 819 | .00 | 17996.88 | 17991.56 | .11 | .10 | .11 | .58 | .56 | .095 |
| 2 Factor: Direct and Indirect | 2271.72 | 818 | .00 | 17894.29 | 17888.92 | .10 | .10 | .11 | .61 | .59 | .094 |
| Bifactor: Direct and Indirect | 1948.05 | 777 | .00 | 17654.62 | 17647.52 | .10 | .09 | .10 | .68 | .65 | .082 |
| 2 Factor: HNE and HSE | 2302.30 | 818 | .00 | 17925.88 | 17920.51 | .10 | .10 | .11 | .60 | .58 | .100 |
| Bifactor: HNE and HSE | 1883.60 | 777 | .00 | 17589.17 | 17582.07 | .09 | .09 | .10 | .70 | .67 | .077 |
| 3 Factor: Direct, Indirect & Spatial | 2230.38 | 816 | .00 | 17857.95 | 17852.50 | .10 | .10 | .11 | .62 | .60 | .100 |
| Bifactor: Direct, Indirect & Spatial | 1789.61 | 777 | .00 | 17495.18 | 17488.08 | .09 | .08 | .09 | .73 | .70 | .078 |
| 4 Factor: Direct HNE, Indirect HNE, Direct HSE, Indirect HSE | 2145.68 | 813 | .00 | 17779.25 | 17773.67 | .10 | .09 | .10 | .64 | .62 | .097 |

Younger cohort, age cut < 6yrs.


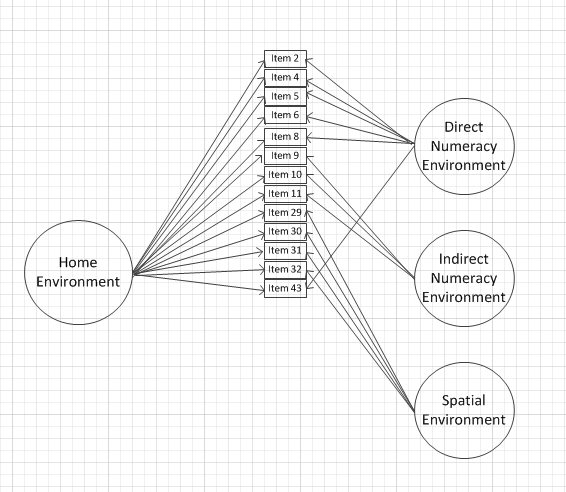


Older cohort, age cut >= 6yrs


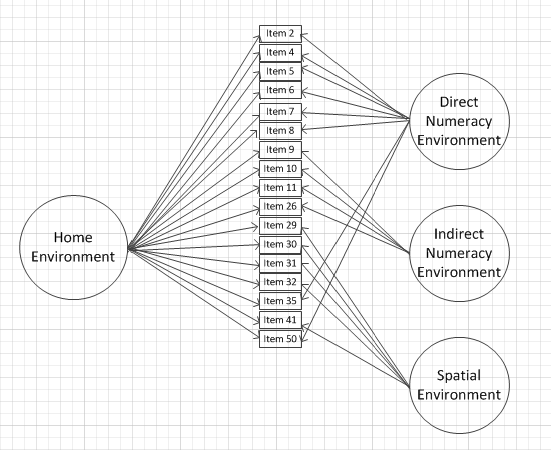


| Model | *χ²* | *df* | *p* | AIC | Adj BIC | RMSEA | RMSEA lower bound | RMSEA upper bound | CFI | TLI | SRMR |
| --- | --- | --- | --- | --- | --- | --- | --- | --- | --- | --- | --- |
| 1 Factor: Home Environment | 2356.73 | 819 | .00 | 17624.77 | 17621.65 | .105 | .100 | .110 | .63 | .61 | .087 |
| 2 Factor: Direct and Indirect | 2236.40 | 818 | .00 | 17506.43 | 17503.29 | .101 | .096 | .106 | .66 | .64 | .087 |
| Bifactor: Direct and Indirect | 1758.44 | 777 | .00 | 17110.48 | 17106.32 | .086 | .081 | .091 | .76 | .74 | .068 |
| 2 Factor: HNE and HSE | 2254.65 | 818 | .00 | 17524.69 | 17521.55 | .101 | .096 | .106 | .65 | .64 | .095 |
| Bifactor: HNE and HSE | 1803.27 | 777 | .00 | 17155.30 | 17151.14 | .088 | .083 | .093 | .75 | .73 | .070 |
| 3 Factor: Direct, Indirect & Spatial | 2180.85 | 816 | .00 | 17454.88 | 17451.69 | .099 | .094 | .104 | .67 | .65 | .095 |
| Bifactor: Direct, Indirect & Spatial | 1707.31 | 777 | .00 | 17059.34 | 17055.18 | .084 | .078 | .089 | .78 | .75 | .069 |
| 4 Factor: Direct HNE, Indirect HNE, Direct HSE, Indirect HSE | 2104.67 | 813 | .00 | 17384.71 | 17381.44 | .096 | .091 | .101 | .69 | .67 | .091 |
